# Effect of elevated body mass index on glycated albumin levels in healthy individuals

**DOI:** 10.1186/s12902-025-02080-2

**Published:** 2025-11-05

**Authors:** Serhat Uysal, Fusun Erdenen

**Affiliations:** 1 Division of Endocrinology and Metabolism, Department of Internal Medicine, Ministry of Health, Burdur State Hospital, Burdur, 15100 Türkiye; 2 Department of Internal Medicine, Istanbul Training and Research Hospital, University of Health Sciences, Istanbul, Türkiye

**Keywords:** Biomarker, Body mass index, Glycated albumin, Healthy individuals, Inflammation

## Abstract

**Background:**

Glycated albumin (GA) is a useful marker for short-term glycemic control, but its levels may be influenced by body composition. Therefore, we aimed to investigate the impact of increasing body mass index (BMI) on GA levels in healthy individuals.

**Methods:**

This cross-sectional study included healthy individuals with normal and elevated BMI. Individuals with diabetes mellitus, pregnancy, acute infection, a history of cardiovascular events, malignancy, chronic liver disease, nephrotic syndrome, thyroid dysfunction, anemia, morbid obesity (BMI ≥ 40 kg/m²), or any other condition known to affect GA levels were excluded. Anthropometric and biochemical measurements were obtained and compared between normal and elevated BMI groups. Statistical analyses were performed using Statistical Package for the Social Sciences (SPSS) version 22.0.

**Results:**

A total of 52 individuals with elevated BMI and 49 with normal BMI were included in the analysis. Individuals with elevated BMI had significantly lower levels of GA (42.8 ± 7.2 vs. 51.3 ± 6.0, *p* < 0.001), while levels of C-reactive protein (CRP) and erythrocyte sedimentation rate (ESR) were markedly higher (0.6 ± 0.4 vs. 0.4 ± 0.2, *p* < 0.001 and 13.5 ± 12.3 vs. 8.3 ± 7.5, *p* = 0.002; respectively). BMI showed a moderate inverse association with GA (*r*=-0.583, *p* < 0.001). Moreover, BMI was positively associated with CRP (*r* = 0.366, *p* < 0.001) and ESR (*r* = 0.299, *p* = 0.002). In addition, GA levels exhibited negative correlations with CRP (*r*=-0.401, *p* < 0.001) and ESR (*r*=-0.384, *p* < 0.001). Multivariate regression analysis confirmed that BMI was independently associated with GA levels (B=-2.727, 95% CI:-5.077 to -0.377, *p* = 0.024).

**Conclusion:**

Our results suggest a potential inverse association between BMI and GA levels.

**Clinical trial number:**

Not applicable.

## Background

Obesity represents a growing global health issue, primarily driven by disrupted energy homeostasis [1–3]. In comparison to individuals with normal weight, those affected by obesity face substantially increased risks of various negative health outcomes [4, 5]. This elevated risk is especially pronounced among diabetic patients, who tend to experience deteriorating glycemic control and worse prognosis as body mass index (BMI) increases [4, 5]. Given these risks, a comprehensive evaluation of the clinical and laboratory implications of obesity remains essential.

Glycated albumin (GA) serves as a reliable indicator of short-term glucose regulation and may provide superior sensitivity compared to glycated hemoglobin A1c (HbA1c), particularly in identifying postprandial hyperglycemia [6–8]. Although GA offers advantages in clinical scenarios where the accuracy of HbA1c may be limited, GA levels can be influenced by inflammatory processes, which may restrict its clinical utility [9–12]. Since chronic low-grade inflammation is commonly associated with obesity, this mechanism has been proposed as a potential explanation for why GA levels may be affected by changes in BMI [11, 12]. These findings raise concerns about the reliability of GA in individuals with diabetes and excess adiposity [11, 12]. Therefore, investigating the impact of BMI on GA levels in metabolically healthy individuals with similar characteristics may help clarify the factors that limit the broader clinical applicability of GA in diabetic populations.

In this study, we aimed to compare GA levels among healthy, non-diabetic individuals and to evaluate their association with BMI. To further explore the possible influence of inflammation on this relationship, we also included acute-phase reactants in the analysis.

## Methods

### Study population

This study included 52 apparently healthy individuals with elevated BMI and 49 with normal BMI who presented to the Internal Medicine outpatient clinics of Istanbul Training and Research Hospital for routine health evaluations or non-specific complaints. All participants were older than 18 years and exhibited adequate cooperation and orientation. Individuals with diabetes mellitus, pregnancy, acute infection, a history of cardiovascular events, malignancy, chronic liver disease, nephrotic syndrome, thyroid dysfunction, anemia, morbid obesity (BMI ≥ 40 kg/m²), or any other condition known to affect GA levels were excluded. To ensure the exclusion of diabetes mellitus, fasting plasma glucose (FPG), HbA1c levels, and participant history were carefully evaluated. Since none of the participants had impaired fasting glucose and the study population consisted of apparently healthy individuals, oral glucose tolerance tests or postprandial glucose measurements were not performed, and therefore, impaired glucose tolerance could not be completely ruled out.

### Anthropometric measurements

Anthropometric and blood pressure assessments were conducted by a single physician (S.U.) to minimize inter-observer variability. Measurements were obtained using standardized, calibrated tools. Standing height was recorded to the nearest 0.1 cm with a stadiometer, and body weight was determined to the nearest 0.1 kg using a digital scale. Blood pressure was assessed in a seated position following a 5-minute resting period, employing an automated sphygmomanometer. Two consecutive measurements were taken at 1- to 2-minute intervals, and the average was used for analysis. BMI was calculated as weight in kilograms divided by the square of height in meters (kg/m²). In accordance with World Health Organization (WHO) criteria, BMI values between 18.5 and 24.9 kg/m² were categorized as normal, and values from 25.0 to 39.9 kg/m² were considered elevated [13].

### Biochemical measurements

Venous blood samples (5 mL) were collected from participants following at least 8 h of overnight fasting. Routine biochemical tests, including FPG, creatinine, alanine aminotransferase (ALT), lipid profile, HbA1c, insulin, albumin, C-reactive protein (CRP), and erythrocyte sedimentation rate (ESR), were performed promptly using fresh blood samples according to standard laboratory protocols. Serum albumin was measured to support the interpretation of GA concentrations, considering that albumin turnover may influence GA levels independently of glycemic control. In addition, spot urine albumin-to-creatinine ratio (UACR) was measured to assess urinary albumin excretion. For GA measurement, an additional aliquot of blood was collected, centrifuged at 3000 rpm at room temperature, and the resulting serum was stored at -80 °C until analysis. GA was measured enzymatically using a colorimetric endpoint method with a commercially available liquid reagent kit (Glycated Serum Protein [Glycated Albumin] Test Kit, Siemens Healthcare Diagnostics Inc., Erlangen, Germany), and results were expressed in µmol/L.

### Statistical analysis

Statistical analyses were carried out using Statistical Package for the Social Sciences (SPSS) Statistics version 22.0. The distribution of continuous variables was assessed with the Shapiro-Wilk test. Variables with normal distribution were reported as mean ± standard deviation (SD), while those without normal distribution were presented as median with interquartile range (IQR). Categorical data were expressed as counts and percentages. For comparisons between two independent groups, the independent samples t-test was used for normally distributed variables, and the Mann-Whitney U test was applied for non-parametric variables. Correlations between continuous variables were evaluated using Pearson’s or Spearman’s correlation tests based on the normality of data. Multivariate linear regression analysis was performed to identify independent predictors of GA levels, adjusting for potential confounders, with results reported as beta coefficients and 95% confidence intervals (CI). A p-value below 0.05 was regarded as statistically significant.

## Results

### General characteristics of the study sample

A total of 101 patients (51 females and 50 males) with a mean age of 29.5 ± 4.8 years were included in the study. The mean BMI was 25.5 ± 4.4 kg/m². FPG was 84.1 ± 9.3 mg/dL, LDL cholesterol was 112.1 ± 31.7 mg/dL, triglycerides were 95.9 ± 57.5 mg/dL, CRP was 0.5 ± 0.3 mg/dL, and ESR was 11 ± 10.5 mm/hour. HbA1c was 5.4 ± 0.3%, GA was 252.0 ± 34.8 µmol/L, and the GA/HbA1c ratio was 46.9 ± 7.9. A detailed summary of the clinical and laboratory characteristics of the study sample is presented in Table 1.

**Table 1:** General clinical and laboratory characteristics of the study sample

Parameters	mean ± SD or *n* (%)
Gender	
Female	51 (50.5)
Male	50 (49.5)
Age (years)	29.5 ± 4.8
BMI (kg/m^2^)	25.5 ± 4.4
Systolic blood pressure (mmHg)	105.5 ± 7.8
Diastolic blood pressure (mmHg)	64.7 ± 7.2
FPG (mg/dL)	84.1 ± 9.3
Creatinine (mg/dL)	0.7 ± 0.2
ALT (IU/L)	19.4 ± 12.5
HDL cholesterol (mg/dL)	50 ± 11.6
LDL cholesterol (mg/dL)	112.1 ± 31.7
Triglyceride (mg/dL)	95.9 ± 57.5
CRP (mg/dL)	0.5 ± 0.3
ESR (mm/hour)	11 ± 10.5
Albumin (mg/dL)	4.5 ± 0.3
Insulin (µIU/mL)	7.4 ± 4.2
UACR (mg/g)	6.4 ± 5.8
HbA1c (%)	5.4 ± 0.3
GA (µmol/L)	252 ± 34.8
GA/HbA1c	46.9 ± 7.9

ALT = Alanine aminotransferase; BMI = Body mass index; CRP = C-reactive protein; ESR = Erythrocyte sedimentation rate; FPG = Fasting plasma glucose; GA = Glycated albumin; HDL = High-density lipoprotein; LDL = Low-density lipoprotein; SD = Standard deviation; UACR = Spot urine albumin-to-creatinine ratio

### Comparison between individuals with normal and elevated BMI

**Fig. 1:**
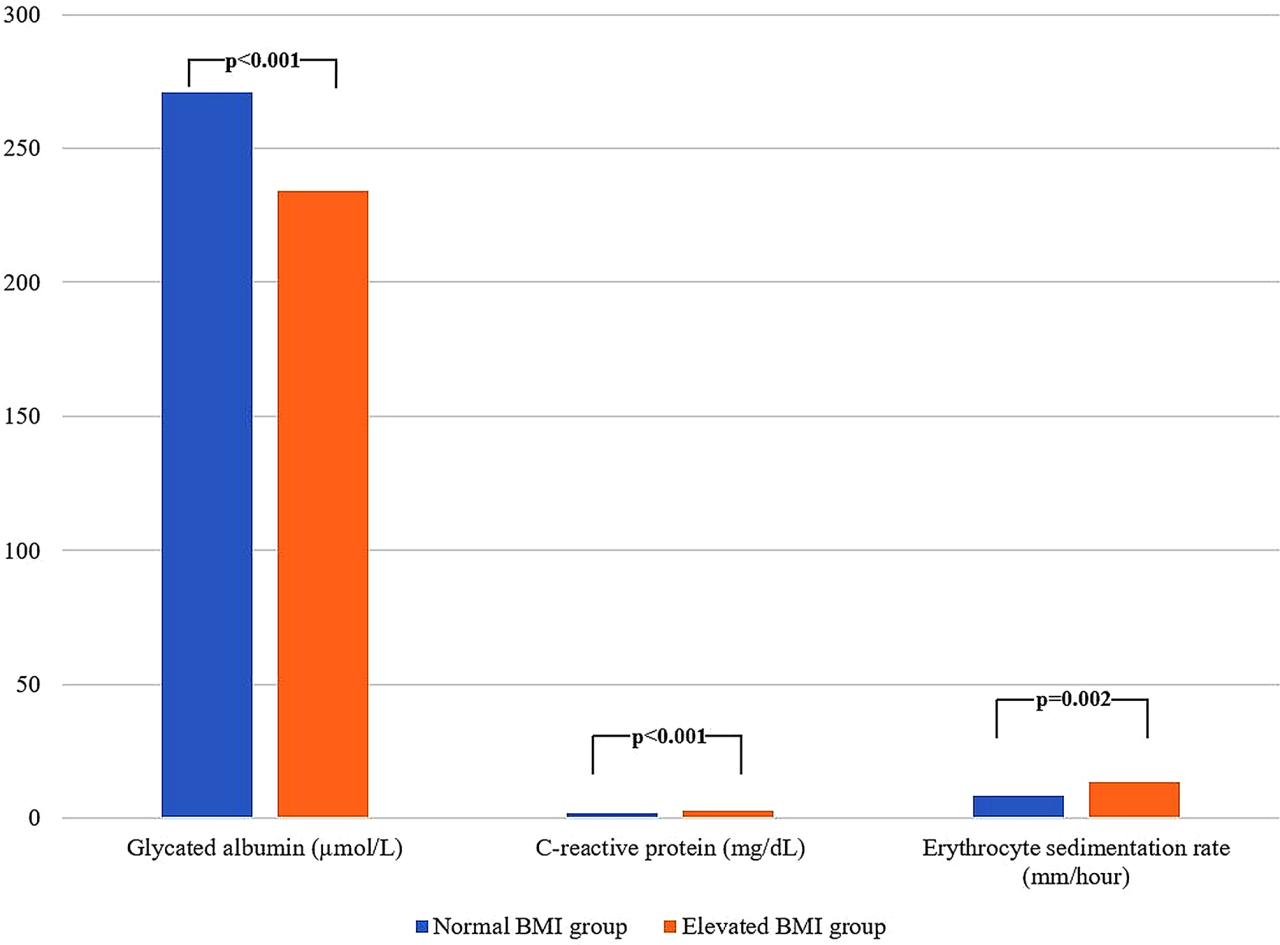
Comparison of glycated albumin, CRP, and ESR between normal and elevated BMI groups

The normal and elevated BMI groups were similar in terms of sex distribution (25 females and 24 males vs. 26 females and 26 males; *p* = 0.918, respectively). Mean age was higher in the elevated BMI group (27.1 ± 2.6 vs. 31.7 ± 5.3, *p* < 0.001). Elevated BMI group also had higher FPG and HbA1c levels (*p* < 0.001 and *p* = 0.002, respectively), whereas GA levels were significantly lower (*p* < 0.001) (Fig. 1). In addition, CRP and ESR were higher in individuals with elevated BMI group (*p* < 0.001 and *p* = 0.002, respectively) (Fig. 1). Detailed biochemical comparisons between the two groups are presented in Table 2.

**Table 2:** Biochemical characteristics according to BMI

Parameters	mean ± SD		p	95% CI of the Difference
	BMI < 25 kg/m^2^ (*n* = 49) ^a^	BMI ≥ 25 kg/m^2^ (*n* = 52) ^b^		
FPG (mg/dL)	79.4 ± 8.1	88.6 ± 8.1	**< 0.001**	[-12.43, -6.02]
Creatinine (mg/dL)	0.7 ± 0.1	0.7 ± 0.2	0.300	[-0.04, 0.07]
ALT (IU/L)	16.1 ± 7.4	22.4 ± 15.3	**0.010**	[-11.10, -1.53]
HDL cholesterol (mg/dL)	53.3 ± 11.4	46.9 ± 11.0	**0.006**	[2.02, 10.89]
LDL cholesterol (mg/dL)	110.1 ± 23.9	114.1 ± 37.7	0.577	[-16.52, 8.59]
Triglyceride (mg/dL)	70.1 ± 23.5	120.2 ± 68.7	**< 0.001**	[-70.54, -29.51]
CRP (mg/dL)	0.4 ± 0.2	0.6 ± 0.4	**< 0.001**	[-0.40, -0.01]
ESR (mm/hour)	8.3 ± 7.5	13.5 ± 12.3	**0.002**	[-9.25, -1.13]
Albumin (mg/dL)	4.6 ± 0.3	4.4 ± 0.3	**0.003**	[0.05, 0.28]
Insulin (µIU/mL)	6.1 ± 2.5	8.5 ± 5.2	**0.003**	[-4.01, -0.78]
UACR (mg/g)	5.3 ± 4.4	7.8 ± 6.4	0.955	[-3.70, 1.31]
HbA1c (%)	5.3 ± 0.2	5.5 ± 0.4	**0.002**	[-0.34, -0.08]
GA (µmol/L)	271.0 ± 27.5	234.1 ± 31.5	**< 0.001**	[25.15, 48.59]
GA/HbA1c	51.3 ± 6.0	42.8 ± 7.2	**< 0.001**	[5.93, 11.18]

ALT = Alanine aminotransferase; BMI = Body mass index; CI = Confidence Interval; CRP = C-reactive protein; ESR = Erythrocyte sedimentation rate; FPG = Fasting plasma glucose; GA = Glycated albumin; HDL = High-density lipoprotein; LDL = Low-density lipoprotein; SD = Standard deviation; UACR = Spot urine albumin-to-creatinine ratio.

Statistically significant values are shown in bold

^a^ BMI values are presented as mean ± SD. In the BMI < 25 group, mean BMI was 21.69 ± 2.07 kg/m²

^b^ BMI values are presented as mean ± SD. In the BMI ≥ 25 group, mean BMI was 29.01 ± 2.64 kg/m²

### Correlation analysis

**Fig. 2:**
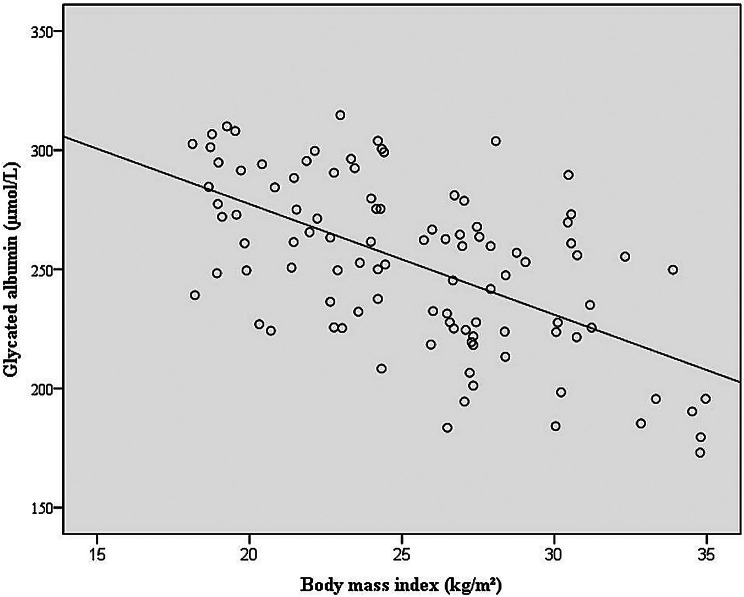
Correlation between glycated albumin and BMI. Illustrates the correlation between glycated albumin levels and body mass index (*r*=-0.583, *p* < 0.001; *n* = 101)

A moderate inverse relationship was observed between BMI and GA levels (*r*=-0.583, *p* < 0.001; *n* = 101) (Fig. 2). Moreover, acute-phase reactants such as CRP and ESR demonstrated a significant upward trend with increasing BMI (*r* = 0.366, *p* < 0.001 and *r* = 0.299, *p* = 0.002; respectively; *n* = 101). GA concentrations were also inversely associated with CRP and ESR values (*r*=-0.401, *p* < 0.001 and *r*=-0.384, *p* < 0.001; respectively; *n* = 101).

Furthermore, higher BMI was linked to lower serum albumin levels (*r*=-0.320, *p* = 0.001; *n* = 101), and showed a positive association with FPG and HbA1c levels (*r* = 0.428, *p* < 0.001 and *r* = 0.349, *p* < 0.001; respectively; *n* = 101).

### Regression analysis

In regression analysis, BMI was independently associated with GA levels (B=-2.727, 95% CI:-5.077 to -0.377, *p* = 0.024), whereas age, sex, CRP, ESR and HbA1c were not significant predictors (all *p* > 0.05). This suggests that higher BMI is linked to lower GA levels in this cohort, independent of demographic, metabolic and inflammatory markers.

## Discussion

Our findings indicate a significant decrease in GA levels with increasing BMI in healthy individuals. Additionally, higher BMI was associated with elevated levels of CRP and ESR, suggesting the presence of a low-grade inflammatory response even in the absence of overt clinical symptoms. This chronic inflammatory state may contribute to the observed reduction in GA levels, as albumin is a negative acute-phase reactant. Regression analysis further confirmed that BMI was independently associated with GA levels. These results underscore the importance of considering BMI as a confounding factor when interpreting GA levels, particularly in individuals with type 2 diabetes, where obesity is highly prevalent. Therefore, the utility of GA as a glycemic marker may be limited in patients with increased adiposity and should be interpreted with caution in this context.

GA has been proposed as a more effective marker of short-term glycemic control than HbA1c, and is also recognized as a valuable tool for both the diagnosis and monitoring of diabetes [14–16]. Prior research involving individuals with type 2 diabetes has shown that GA concentrations tend to be reduced in obese patients relative to those without obesity [12, 17]. In our study, an inverse association between GA levels and BMI was identified, independent of diabetes presence or sex. Similarly, He et al. reported results consistent with our findings [18]. On the other hand, Nishimura et al. observed lower GA levels in obese children [19]. However, a retrospective analysis of clinical trial data by Powers Carson et al. did not detect a significant negative correlation between GA and BMI across a wide BMI range, although weak negative correlations were observed between BMI and glycated serum proteins, albumin, and the GA/HbA1c ratio. The authors suggested that inflammation, insulin-mediated alterations in protein synthesis, or BMI-related changes in renal clearance could partly account for these findings [20]. To further clarify the independent contribution of BMI to GA levels in our cohort, we performed multivariate regression analysis including age, sex, CRP, ESR, and HbA1c as covariates. This analysis confirmed that BMI was independently associated with GA, whereas the other variables were not significant predictors, reinforcing the robustness of our findings. Observations from diverse populations generally support our findings and highlight the importance of interpreting GA levels with caution in individuals with elevated BMI. Moreover, another study proposed that differences in the rate of GA synthesis may also contribute to the lower GA levels observed in individuals with elevated BMI [21]. Taken together, these data suggest that increased adiposity may influence GA concentrations, which should be taken into account when using GA as a glycemic marker in clinical practice.

Another notable finding of our study was the negative correlation between GA levels and acute-phase reactants, including CRP and ESR. Both CRP and ESR were positively associated with higher BMI, supporting the presence of a low-grade inflammatory state in individuals with increased adiposity. This inflammation may contribute to reduced GA levels through catabolic effects on albumin metabolism and decreased hepatic albumin synthesis, potentially mediated by proinflammatory cytokines, as previously suggested in the literature [22]. Consistent with our observations, Koga et al. reported that obesity and obesity-related chronic inflammation were associated with decreased GA levels in non-diabetic individuals [23]. Moreover, recent data indicate that inflammatory cytokines, including IL-10, may play a role in modulating the relationship between GA and BMI, further supporting the contribution of inflammation to GA variations in individuals with elevated BMI [24]. Another hypothesis proposes that increased BMI may lead to glomerular hypertrophy over time by elevating glomerular filtration rate, which could contribute to obesity-related glomerulopathy [25, 26]. However, in the present study, no significant differences in UACR were observed across BMI categories. This finding may be explained by the relatively small sample size and the possibly insufficient duration of exposure to elevated BMI required for the development of obesity-related glomerulopathy.

Our results demonstrate that individuals with higher BMI exhibited elevated FPG and HbA1c levels. This result aligns with previous studies conducted in non-diabetic populations and may reflect the increased insulin resistance and impaired glucose metabolism associated with obesity [18, 27]. All these results support the notion that BMI has a differential effect on various glycemic markers and underscore the complex interplay between body composition and glucose regulation.

It is important to acknowledge several limitations of this study. First, its cross-sectional design precludes any inference of causal relationships. Second, the relatively small sample size, without a priori power analysis, may limit the generalizability of the findings and reduce the statistical power of subgroup analyses. Additionally, other potential confounding factors, such as diet, physical activity, and socioeconomic status, were not assessed, which may have influenced the observed associations. Third, the study employed WHO BMI cutoffs (25.0–39.9 kg/m²). While lower BMI thresholds are sometimes recommended for certain Asian populations due to higher metabolic risk at lower BMI, our cohort consisted of Turkish adults, whose BMI-related metabolic risk is generally comparable to European populations; therefore, the WHO cutoffs are considered appropriate. Furthermore, the lack of longitudinal follow-up limits the ability to evaluate temporal changes in GA, CRP, or ESR levels in relation to BMI variations. Finally, as the study population primarily included young Turkish adults, caution should be exercised when extrapolating these findings to older individuals, children, or non-Turkish populations.

## Conclusions

Our findings suggest that higher BMI is associated with lower GA levels. This association should be taken into account when interpreting GA as a glycemic marker, particularly in individuals with diabetes and elevated BMI.

## Data Availability

The data archive can be made available on request. Further requests can be directed to the corresponding author.

## Abbreviations

ALT: Alanine aminotransferase
BMI: Body mass index
CI: Confidence interval
CRP: C-reactive protein
ESR: Erythrocyte sedimentation rate
FPG: Fasting plasma glucose
GA: Glycated albumin
HbA1c: Glycated hemoglobin A1c
IQR: Interquartile range
LDL: Low-density lipoprotein
SD: Standard deviation
SPSS: Statistical package for the social sciences
UACR: Spot urine albumin-to-creatinine ratio
WHO: World health organization
